# Microneme-located VP2 in *Eimeria acervulina* elicits effective protective immunity against infectious bursal disease virus

**DOI:** 10.1128/iai.00456-23

**Published:** 2024-01-05

**Authors:** Ying Yu, Xinming Tang, Chunhui Duan, Jingxia Suo, Colin Crouch, Sixin Zhang, Xianyong Liu, Jie Liu, Beth Bruton, Ian Tarpey, Xun Suo

**Affiliations:** 1 National Key Laboratory of Veterinary Public Health and Safety, Beijing, China; 2 Key Laboratory of Animal Epidemiology and Zoonosis of Ministry of Agriculture, Beijing, China; 3 National Animal Protozoa Laboratory & College of Veterinary Medicine, China Agricultural University, Beijing, China; 4 Key Laboratory of Animal Biosafety Risk Prevention and Control (North) of MARA, Institute of Animal Sciences, Chinese Academy of Agricultural Sciences, Bejing, China; 5 MSD Animal Health, Walton Manor, Milton Keynes, United Kingdom; University of Pennsylvania, Philadelphia, Pennsylvania, USA

**Keywords:** *Eimeria acervulina*, surface, microneme, immune responses, viral antigen

## Abstract

Using transgenic *Eimeria* spp. to deliver exogenous antigens is a viable option for developing multivalent live vaccines. Previous research revealed that the location of antigen expression in recombinant *Eimeria* dictates the magnitude and type of immune responses. In this study, we constructed genetically modified *Eimeria acervulina* that expressed VP2 protein, a protective antigen from infectious bursal disease virus (IBDV), on the surface or in the microneme of sporozoites. After vaccination, VP2-specific antibody was readily detected in specific pathogen-free chickens receiving transgenic *E. acervulina* parasites expressing VP2 in microneme, but animals vaccinated with which expressing VP2 on surface failed to produce detectable antibody after two times immunizations. Moreover, the bursal lesion of microneme-located VP2 transgenic *E. acervulina* immunized chickens was less severe compared with un-immunized animals after IBDV challenge infection. Therefore, genetically modified *E. acervulina* that express IBDV-derived VP2 in micronemes are effective in inducing specific antibody responses against VP2, while parasites that have VP2 expression on cell surface are not suitable. Thus, the use of *Eimeria* parasites as vaccine vectors needs to consider the proper targeting of exogenous immunogens. Our results have implications for the design of other vector vaccines.

## INTRODUCTION

Poultry production is a vital way to reduce poverty and enrich global food supplies (1, 2). Huge demands have led to a rapid expansion of the poultry industry, but the resulting high-density poultry farming is accompanied by the risk of infectious disease outbreaks (3). Vaccination has been the most practical method for controlling infectious diseases, and chickens are more intensively vaccinated than other livestock animals (4, 5). These vaccines are usually used to protect from viral pathogens, including avian influenza virus, Newcastle disease virus, infectious bronchitis virus, and infectious bursal disease virus (IBDV).

Chicken coccidiosis is a huge economic burden to the poultry industry as well (6), and seven causal *Eimeria* species (*E. tenella*, *E. acervulina*, *E. maxima*, *E. necatrix*, *E. mitis*, *E. brunetti,* and *E. praecox*) are known to differ in pathogenicity, reproduction, immunogenicity, and other biological characteristics (7, 8). Certain wild-type/attenuated *Eimeria* oocysts serve as live vaccines for the control of chicken coccidiosis (9, 10), while genetic engineering can also convert these single-celled parasites to vectors that deliver exogenous antigens of other pathogens, as exemplified by successes in stable transfection in *E. tenella*, *E. acervulina*, *E. necatrix,* and *E. mitis* (11–14). These transgenic *Eimeria* are able to induce immune responses against *Eimeria* antigens, but the magnitude and type of immune responses against exogenous antigens of bacterial or viral origins are often inconsistent, suggesting that not all these species are suitable for use as live vaccine vectors. Given the need for optimizing genetic manipulation strategies for *Eimeria* species as vaccine vectors, we envisioned that *E. acervulina* and *E. mitis* are safer than *E. tenella* and *E. necatrix* as both have low pathogenicity in chickens. Meanwhile, although *E. mitis* is less pathogenic than *E. acervulina*, the latter is more practical as it is more prevalent in the field (15) and commonly used in oocysts-based live vaccines against coccidiosis. Extensive research on *E. acervulina*, including the selection of precocious and serotinous lines, high-quality and high-resolution genome assembly, as well as the optimization of genetic hybridization system (16), have resulted in an in-depth knowledge of *E. acervulina*. Therefore, *E. acervulina*, with its high immunogenicity (17), holds greatest potential as a carrier for multivalent vaccines when compared with three other sibling species.

Infectious bursal disease (IBD), caused by IBDV infection, is an acute and highly contagious disease that significantly affects the poultry industry (18). IBDV tends to infect 3- to 6-week-old chickens and destroy immature B cells in the bursa of Fabricius, which causes immunosuppression that not only predisposes chickens to a variety of diseases but also leads to vaccination failure (19). Therefore, it is necessary to use vaccines to prevent IBD at an early age of chickens. Many types of IBDV vaccines have been developed, including conventional live and inactivated vaccines, immune complex vaccines, live viral vector vaccines, subunit vaccines, and DNA vaccines (20). However, the high variability of IBDV makes new requirement of vaccines to resist IBD. VP2 protein is the main structural protein of IBDV determining variability, and the main protective antigen that stimulates host protective immunity (21), so it is the focus of subunit vaccine and recombinant vaccine development of IBDV.

The location of exogenous antigens in the subcellular components of genetically modified *Eimeria* may determine the magnitude and type of immune responses. In the present study, we have used *E. acervulina* as an expression system to generate transgenic *Eimeria* strains expressing the VP2 antigen of IBDV at two subcellular locations, microneme or surface, and compare the immune responses induced by the two strains of transgenic *E. acervulina*.

## RESULTS

### Construction of transgenic *E. acervulina* expressing IBDV-derived VP2 in different subcellular compartments

**Fig 1:**
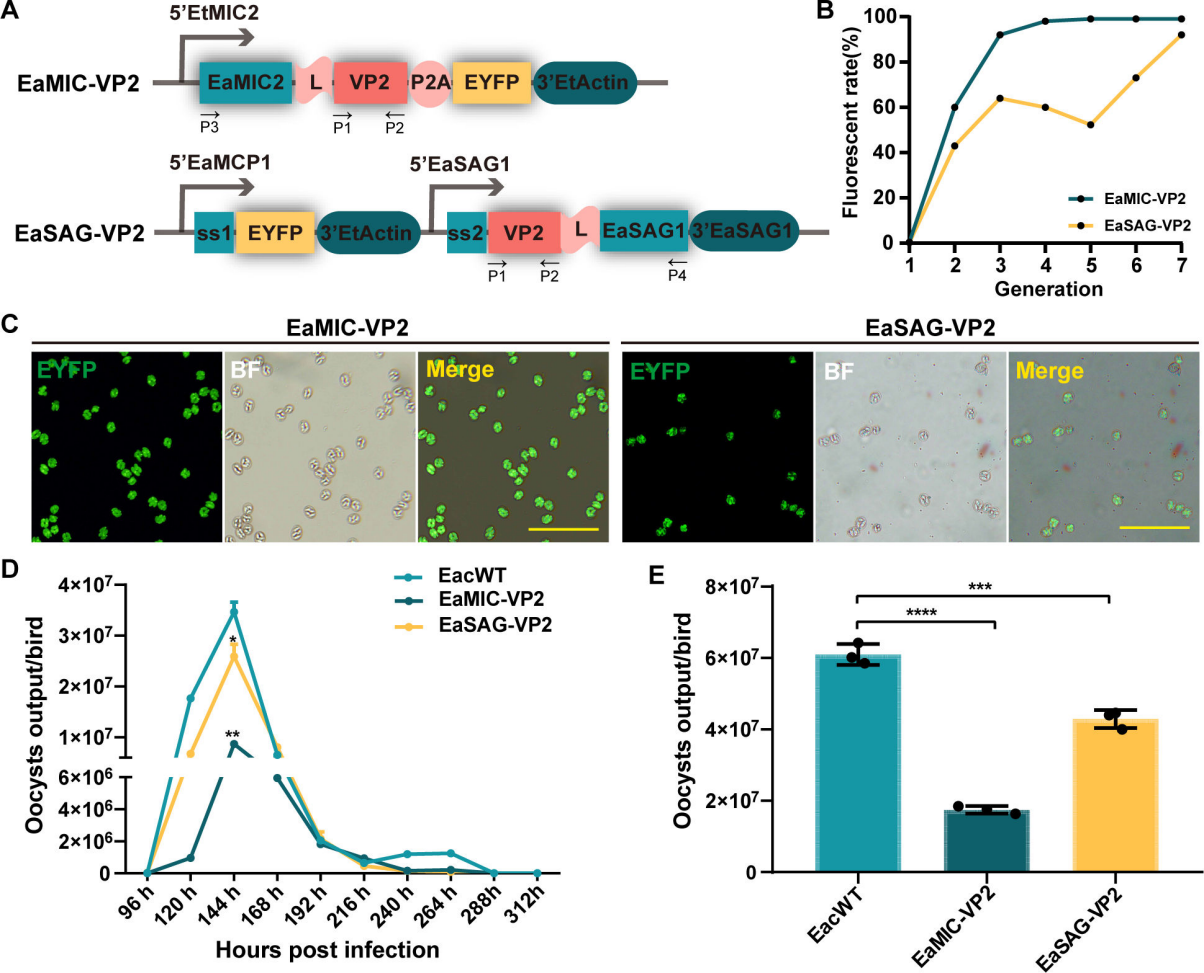
Construction of transgenic *E. acervulina* expressing IBDV-derived VP2. (**A**) Schematic of constructs for transgenic *E. acervulina* expressing VP2. Construct pEaMIC-VP2, in which EYFP was fused to the C-terminal of EaMIC2, for expressing VP2 in microneme. Construct pEaSAG-VP2, in which EYFP was fused between the signal peptide and mature peptide of EaSAG1, for expressing VP2 on the surface of sporozoites. ss1, signal sequence of EaMCP1; ss2, signal sequence of EaSAG1. (**B**) Passage and fluorescent tracking of transgenic strains before obtaining progeny from single sporocyst isolation. (**C**) Fluorescence microscopy of transgenic progeny from single sporocyst isolation. The intensity was consistent in both populations of sporulated oocyst. Bar = 100 µm. (**D**) Oocysts output curve of wild-type and transgenic *E. acervulina* on free-coccidiosis Arbor Acre (AA) broilers. (**E**) Total oocysts output of different stains at 5–8 days. Asterisks represent for statistical difference: * stands for *P* < 0.05, ** stands for *P* < 0.01, *** stands for *P* < 0.001, **** stands for *P* < 0.0001.

Two plasmids, pEaMIC-VP2 (VP2 directed to the microneme) and pEaSAG-VP2 (VP2 directed to cell surface) were constructed for stable transfection in *E. acervulina* (Fig. 1A). After fluorescence-activated cell sorting (FACS) selection and propagation of transfected *E. acervulina*, we obtained two enhanced yellow fluorescent protein (EYFP) positive strains with fluorescent rate greater than 95% (Fig. 1B). For single sporocyst isolation, we acquired 1 of 11 clones for EaMIC-VP2 and 1 of 13 clones for EaSAG-VP2, respectively. Both populations of sporulated oocyst were expanded for propagation (Fig. 1C). The oocyst shedding curve showed that the transgenic strains had the same pattern, including patent period and peak period, but the oocyst output at 144 hours after inoculation, i.e., peak period, decreased significantly than the EacWT (Fig. 1D). The total number of oocysts output between 5 to 8 days after inoculation showed clearly that transgenic strains have lower fecundity, especially EaMIC-VP2 (Fig. 1E).

### The transcription and expression of VP2 gene in transgenic parasites

**Fig 2:**
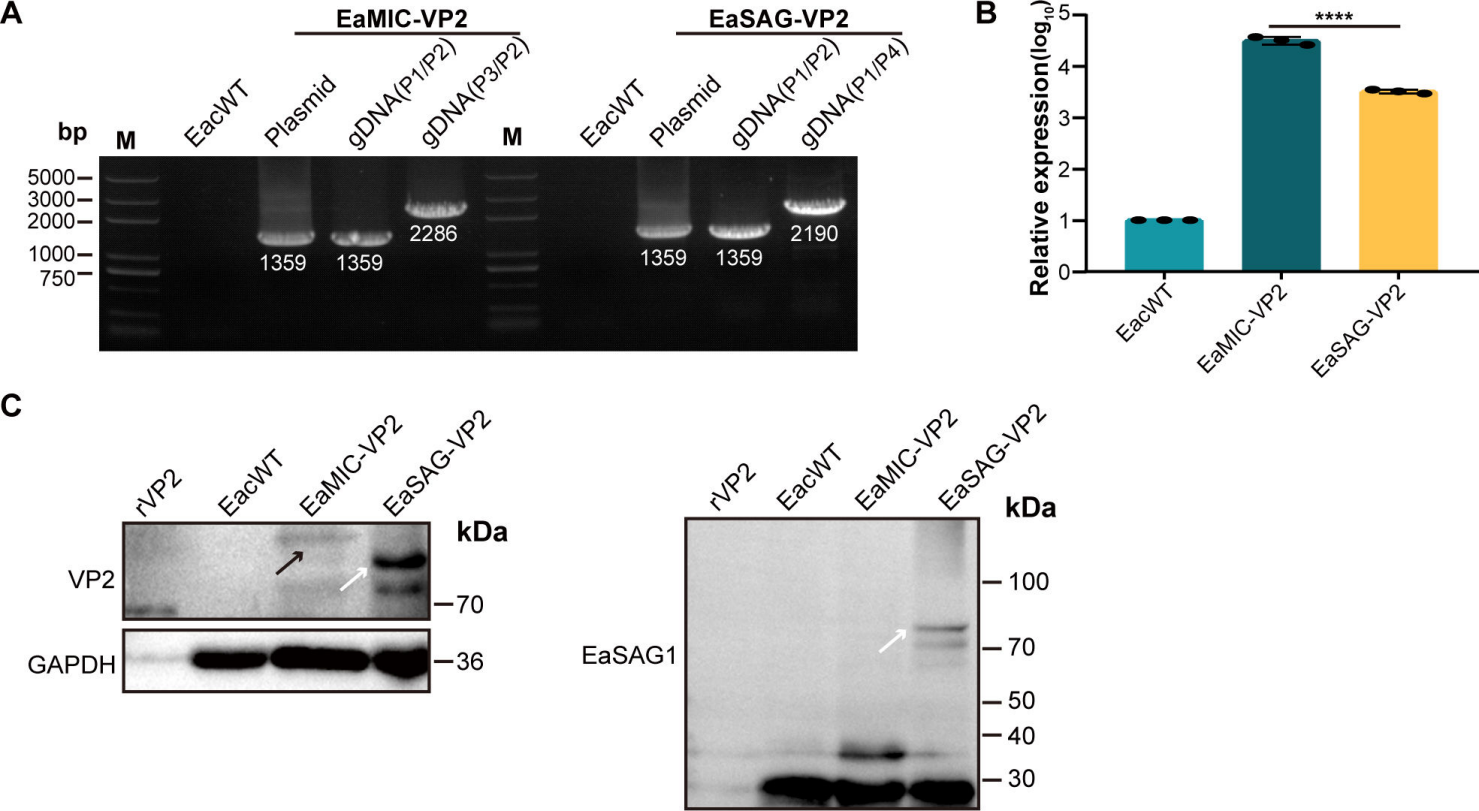
Analysis of VP2 in transgenic *E. acervulina* strains at the gDNA, mRNA, and protein levels. (**A**) Identification of target gene *vp2* in the genome of the transgenic strains by PCR. The identification primers were shown in Fig. 1A and the target fragments were 1,359 bp for P1/P2, 2,286 bp for P3/P2, and 2,190 bp for P1/P4, respectively. The genome (gDNA) of EacWT was used as the negative control template, and the transfected plasmids were used as the positive control templates; M, marker. (**B**) The relative transcript level of *vp2* in transgenic strains. Glyceraldehyde-3-phosphate dehydrogenase (GAPDH) was used as the internal control in the RT-qPCR analysis. (**C**) Identification of VP2 expression in the whole soluble protein of transgenic *E. acervulina* by Western blot. The anti-VP2 and anti-EaSAG1 antibodies were employed to detect the expression of VP2, which was observed as fusion proteins with EaMIC2 (indicated by the black arrow) and EaSAG1 (indicated by the white arrow) in transgenic *E. acervulina* strains. M, marker.

The presence and expression of VP2 sequences in the two transgenic *E. acervulina* was confirmed at genomic DNA (gDNA), mRNA, and protein levels. The *vp2* gene was shown to exist in the genome of transgenic parasites by PCR analysis (Fig. 2A). Real-time fluorescent quantitative PCR (RT-qPCR) showed that the relative transcript level of *vp2* in EaMIC-VP2 was 10 times or so as high as in EaSAG-VP2 (Fig. 2B). Western blot indicated that VP2 indeed existed in the form of fusion proteins with EaMIC2 or EaSAG1 in the two transgenic parasites, respectively (Fig. 2C).

### Subcellular location of VP2 in transgenic *E. acervulina* strains

**Fig 3:**
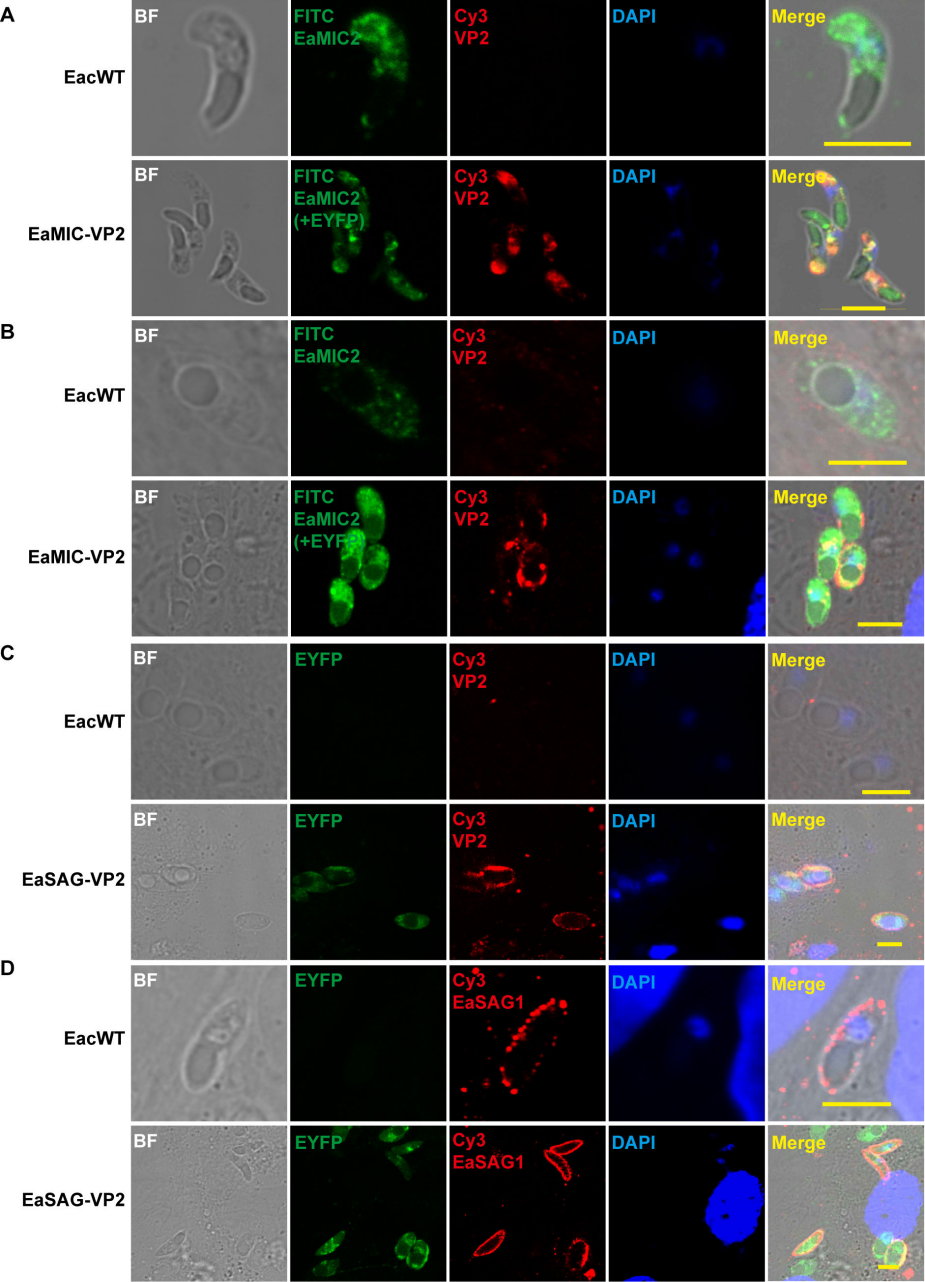
Location of VP2 proteins in transgenic *E. acervulina*. (**A** and **B**) VP2 expression form in sporozoites of transgenic strain EaMIC-VP2. Sporozoites was identified extracellularly (**A**), or intracellularly (**B**): VP2 was colocalized with the microneme protein EaMIC2 in sporozoites. (**C** and **D**) VP2 expression form in sporozoites of transgenic strain EaSAG-VP2. Both VP2 (**C**) and EaSAG1 (**D**) were identified as surface location of intracellular transgenic sporozoites. Bar = 5 µm.

Indirect immunofluorescence assay (IFA) was used to identify the location of VP2 in the two transgenic *E. acervulina*. In EaMIC-VP2 parasites, VP2 was located at the apical end of sporozoites in cell-free culture conditions (Fig. 3A) but dispersed across the surface upon invading cells when cultured with human foreskin fibroblast (HFF) cells (Fig. 3B). In EaSAG-VP2 parasites, VP2 exhibited a sporadic surface expression pattern on intracellular sporozoites (Fig. 3C), where EaSAG1 was localized (Fig. 3D).

### The transgenic strains elicited good antibody response against *E. acervulina* and detectable antibody titer against IBDV

**Fig 4:**
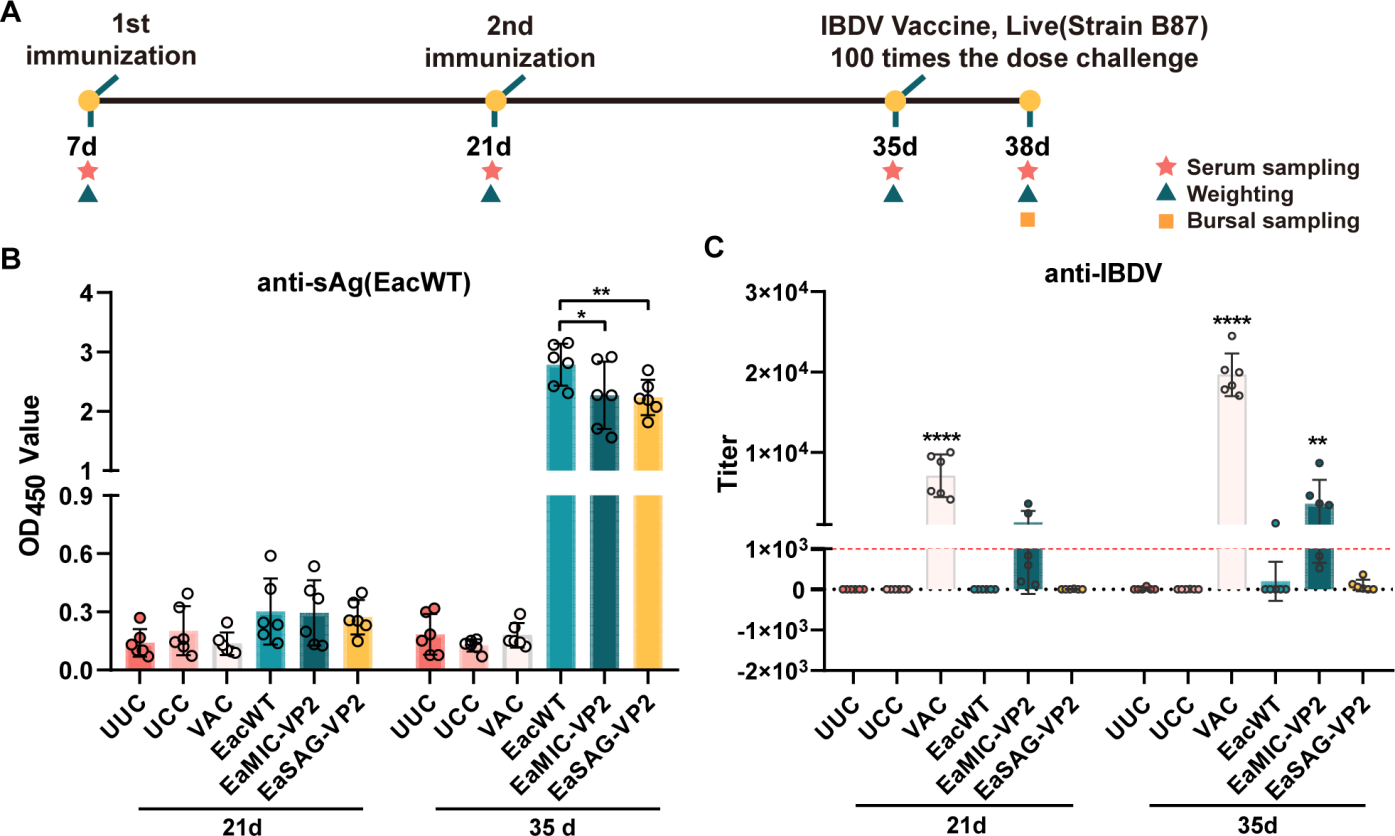
Comparison of antibody responses elicited by transgenic strains expressing IBDV-derived VP2. (**A**) Diagram of immunological experiments. The first and second immunization were performed at the age of 7 days (7d) and 21 days (21d) old on SPF chickens, respectively; immunization doses were operated as shown in Table 1. *n* = 6. (**B**) Antibody levels against EacWT sAg in SPF chicken serum, detected by OD_450_. (**C**) Antibody titers against IBDV in SPF chicken serum. The assay was performed using the ProFLOK IBD PLUS Ab Test kit, and the red dashed line represents a titer equal to 999, values greater than which are considered positive.

The immunological experiment was performed on specific pathogen-free (SPF) chickens according to the schedule (Fig. 4A; Table 1). The antibody titer against soluble antigen (sAg) of EacWT showed good immunity against *Eimeria*, but both of transgenic strains were not as good as EacWT (Fig. 4B). Serum antibody against IBDV were detected using two kits: both kits showed high titers of group in which chickens were immunized by commercial recombinant VP2 (rVP2) vaccine (VAC group); the ProFLOK IBD PLUS Ab Test kit showed more positive values in the group immunized by EaMIC-VP2 parasites while the other kit detected scarcely positive values in groups except VAC group; chickens in the group immunized with EaMIC-VP2 parasites (EaMIC-VP2 group) showed higher titer values after the second immunization than the first immunization (Fig. 4C; Fig. S1A), although there was no significant difference of bursal index among all groups 3 days after IBDV challenge (Fig. S1B).

**TABLE 1:** Groups, immunization doses, and IBDV challenge in the immunological test^*c*^

Group	Immunization dosage		Challenge
	First	Second	
UUC	PBS^*a*^	PBS^*a*^	PBS^*a*^
UCC	PBS^*a*^	PBS^*a*^	IBDV
VAC	300 µL^*b*^	PBS^*a*^	IBDV
EacWT	1 × 10^4^	5 × 10^5^	IBDV
EaMIC-VP2	1 × 10^4^	5 × 10^5^	IBDV
EaSAG-VP2	1 × 10^4^	5 × 10^5^	IBDV

^*a*^ Inoculated orally 200 μL PBS for chickens. PBS, phosphate buffer saline.

^*b*^ Inoculated intramuscularly 300 μL for 7- to 14-day-old chickens, the immune duration reached 4 months, as described by the manufacturer.

^*c*^ UUC, un-immunized and unchallenged control; UCC, un-immunized and challenged control; VAC, immunized intramuscularly with the inactivated tetravalent vaccine containing IBDV rVP2; EacWT, immunized orally with the sporulated oocysts of EacWT suspended in 200 μL PBS; EaMIC-VP2, immunized orally with the sporulated oocysts of EaMIC-VP2 suspended in 200 μL PBS; EaSAG-VP2, immunized orally with the sporulated oocysts of EaSAG-VP2 suspended in 200 μL PBS.

### Immune protection in chickens immunized with the transgenic strain EaMIC-VP2

**Fig 5:**
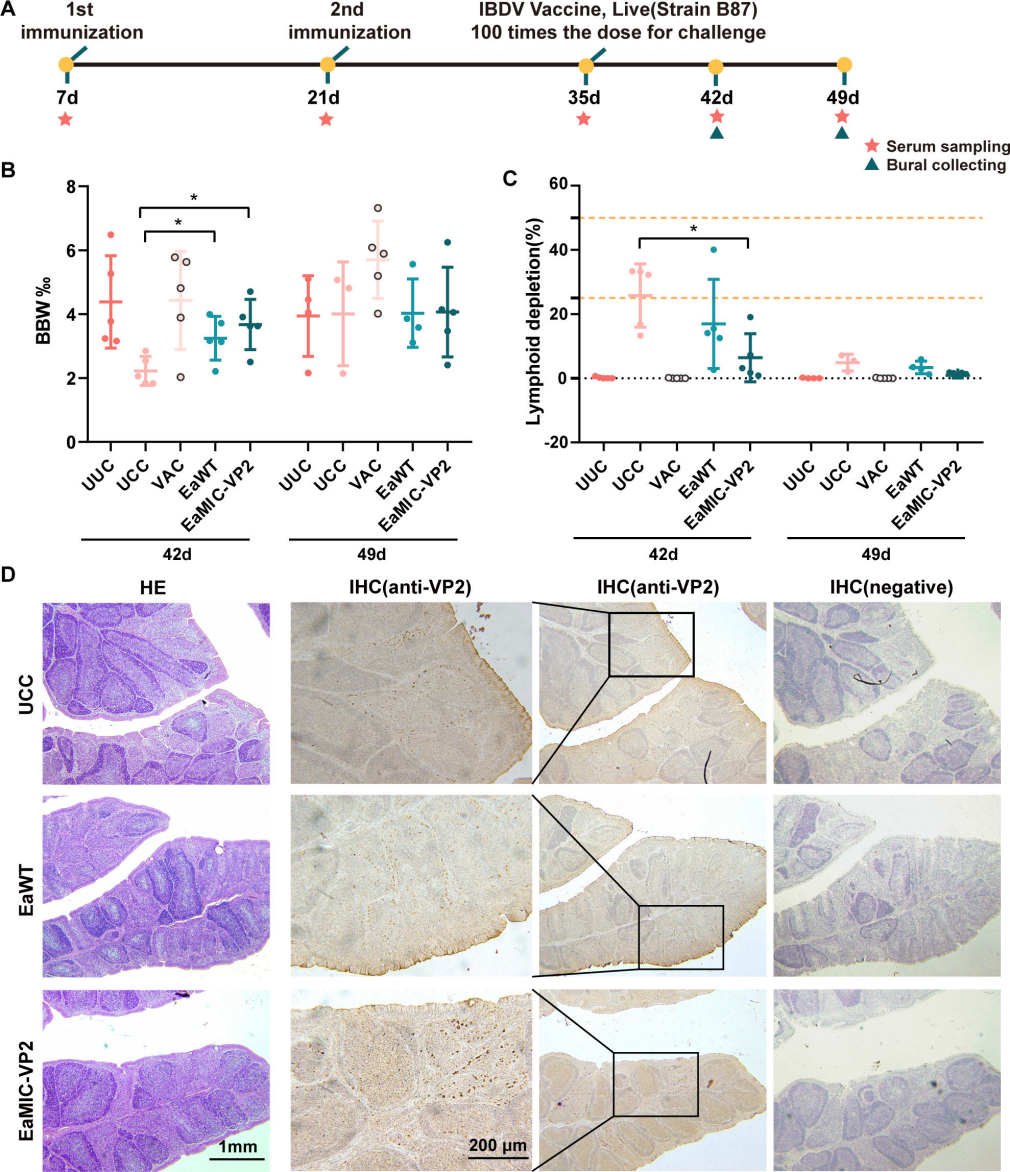
The protect effect of transgenic strain EaMIC-VP2 against IBDV challenge. (**A**) Diagram of immunological experiments. The first immunization was performed on 7-day-old SPF chicken, the other immunization and challenge were performed at 14-day intervals successively. The immunization doses are shown in Table 1. The SPF chickens except UUC group were challenged by 100-fold dose of live IBDV vaccine (B87 strain) at 42d. The bursas of Fabricius were weighed and collected at 42d and 49d after challenge. *n* = 5. (**B**) BBW at 7 (42d) and 14 days (49d) after challenge. (**C**) Lymphoid depletion of bursal evaluated by pathological section. (**D**) Pathological sections and immunohistochemical (IHC) staining of bursal at 42d. IHC staining showed the positivity for tissue damage that was typical of IBDV infection in hematoxylin and eosin (HE) staining sections.

Considering the moderate virulence of IBDV which was used for challenge, bursal were sampled at two later timing, 7 days (42d) and 14 days (49d) after IBDV challenge (Fig. 5A). The immune organ index was calculated as bursal/body weight ratio (BBW), which showed less bursal weight loss of chickens in EaMIC-VP2 group than un-immunization and challenged control (UCC) group at 42d, but the loss could recover at 49d (Fig. 5B). Besides, the lymphocyte depletion rate (Fig. 5C) used to evaluate the lesion score (Table 2) was observed by bursal hematoxylin and eosin (H&E) staining sections (Fig. S2) and showed consistent results with BBW. Furthermore, bursal lesions were typical of IBDV infection (Fig. 5D).

**TABLE 2:** Bursal lesion score as measured on day 7 after IBDV challenge infection^*a*^

Group (*n* = 5)	Bursal lesion score of individual chicken	Average (mean ± SD)
UUC	0, 0, 0, 0, 0	0 ± 0.000
UCC	1, 1, 2, 2, 2	1.6 ± 0.548
VAC	0, 0, 0, 0, 0	0 ± 0.000
EacWT	1, 1, 1, 1, 2	1.2 ± 0.447
EaMIC-VP2	0, 1, 1, 1, 1	0.8 ± 0.447

^*a*^ Note: the score is a semi-quantitative measure: 0 = no lesion, 1 = 1%–25%, 2 = 26%–50%, 3 = 51%–75%, 4 = 76%–100% of follicles showing more than 50% lymphocyte depletion.

## DISCUSSION

Our work here builds on our recent development of a series of genetically engineered *E. acervulina* (14, 16). For the two transgenic *E. acervulina* strains which correctly targeted VP2 of IBDV to specific subcellular locations (microneme versus cell surface), microneme-located VP2 was immunogenic enough to induce specific antibody responses against the IBDV, but the surface-located VP2 was suboptimal, in immunized animals. These results lend further support for the notion that *Eimeria* parasites may serve as multivalent vaccine vectors when exogenous immunogens are targeted to the right subcellular compartment.

The construction of stable transgenic *Eimeria* strains is the first step to develop live vaccines based on *Eimeria*. The fluorescent efficiency of *Eimeria* transfection mediated by restriction enzyme-mediated integration (REMI) is usually in a few thousandths and considered unstable with fluorescent selection only, and so is always selected doubly with drug pyrimethamine and FACS (5, 14, 22, 23). However, we constructed two stable transgenic populations without drug-resistant genes but with high fluorescence selecting efficiency (Fig. 1A and B), which could avoid drug resistance in field chicken populations for appropriate live vaccines. Besides, we isolated clonal populations by single sporocyst infection of chickens (Fig. 1C). Though the process was time-consuming and inefficient, the acquired clonal populations facilitated the study of characteristics for transgenic *E. acervulina*. Transgene *vp2* was shown the integration and transcription in both transgenic populations (Fig. 2A and B), which had been also reported before. Additionally, we identified directly here the expression of VP2 and fusion proteins with VP2 by liquid chromatography tandem mass spectrometry (LC-MS/MS) and Western blot, respectively (Fig. S3 and Fig. 2C), which was only identified by indirect methods before (5). Therefore, the two transgenic *E. acervulina* provided us materials for further study.

Good immune responses are necessary for *Eimeria* to be used as multi-valent vaccines. Here, we analyzed preliminarily the antibody responses stimulated by transgenic *E. acervulina* expressing viral proteins, VP2 of IBDV, in two distinct cellular compartments (Fig. 4). Microneme-located VP2 showed stronger immunogenicity than surface-located VP2, but there is still some way to go compared with recombinant VP2 in commercial vaccine. Marugan-Hernandez et al. (5) proposed that optimization of the number of parasites per vaccinating dose or transference of the technology to more fecund *Eimeria* species might encourage stimulation of more significant immune response, after they detected low level of responses induced by *E. tenella*-delivered viral proteins in chicken immune system. *E. acervulina* used in our study had the characteristic with the high fecundity and low pathogenicity. Thus, we performed another immunological experiment with a double dose of oocysts for the first immunization, and we found higher and more detectable antibody titer after immunizations than before (Fig. 4C and Fig. S4), and some values of which in EaMIC-VP2 group were comparable to that in the VAC group. The oral immunization of EaMIC-VP2 parasites demonstrated the potential to prime immunity, as evidenced by higher antibody titers observed in the EaMIC-VP2 group compared to the UCC group at 7 days (42d) after IBDV challenge. In addition, the specific antibody response stimulated by microneme-located VP2 could resist the challenge of moderate IBDV to a certain extent, which was identified by immunohistochemistry (IHC) (Fig. 5D). The results confirmed that enhancing the total amount of foreign antigens expressed by *Eimeria* was vital to optimize the antibody responses. Interestingly, while microneme location was initially believed to have no impact on humoral immune response in contrast to cytoplasmic location (24), it remarkably displayed a significant advantage in IBDV-specific antibody response over surface location.

Previous studies have indicated that the location of immunogens does impact the antigen-presenting pathways during immune surveillance (25–27). Cell surface proteins of apicomplexan parasites have the advantage of exposing and attaching to host cells first (28); as such, immunogens present on the surface of transgenic *Eimeria* are expected to be more readily recognized by immune system than their internal counterparts in promoting antibody responses, which is contrary to results obtained from our study here, as animals vaccinated with parasites expressing VP2 on their cell surface failed to produce detectable antibodies. The most likely explanation is that the VP2 antigens displayed on cell surface were subject to proteinase-mediated degradation. The sporozoites were harvested from sporulated oocysts after *in vitro* excystation with bile-trypsin, and this step can lead to partial VP2 degradation. Additionally, IFA used for identifying intracellular sporozoites showed only sporadic staining on the surface (Fig. 3), indicating that VP2 antigens displayed on the surface indeed underwent partial degradation. Thus, there is a clear trade-off between surface expression and degradation in vaccine development.

For the future further study, the enhancement of immune response induced by *Eimeria*-based VP2 is necessary. On the one hand, to improve the quantity of VP2 in *E. acervulina*, increasing the number of copies of VP2 in EaMIC-VP2 parasites or/and simultaneously targeting VP2 into multiple organelles in *E. acervulina* could be considered, increasing the times or/and dose of sporulated oocysts for vaccination. CRISPR-mediated transcriptional activation system (29) might be also a choice. On the other hand, to enhance the ability of microneme-located VP2 to stimulate immune response, molecular adjuvants (30), like immunomodulatory factor (31) and bacterial-derived subunits (32) with immune-stimulating effects, could be fused by genetic manipulation. The plasmids and clonal parasites produced in this study provide materials for exploring effective strategies.

## MATERIALS AND METHODS

### Parasites and animals

*Eimeria acervulina* (BJ strain) used in this study came from our laboratory at China Agricultural University. Procedures for the collection, purification, and sporulation of oocysts have been fully described (33).

One-day-old coccidian-free broiler chickens were purchased from Beijing Arbor Acres Poultry Breeding Co., Ltd. They were kept in a coccidian-free and air-conditioned poultry isolator and given adequate feed and water. Experiments with these animals began when they were 1 week old.

One-week-old SPF chickens were purchased from Beijing Merial Vital Laboratory Animal Technology Co., Ltd. They were fed a pathogen-free diet and water *ad libitum*.

### Reagents and antibodies

A tetravalent vaccine against Newcastle disease, infectious bronchitis, avian influenza (subtype H9), and infectious bursal disease (using inactivated strain La Sota + strain M41 + strain SZ + protein rVP2) (Pulike Biological, China) was provided by Dr. Liangquan Zhu (China Institute of Veterinary Drug Control, Beijing, China.) and kept at 4°C until use. The infectious bursal disease vaccine (live strain 87) (YIBIO, China) was stored at −20°C.

The polyclonal antibodies, mouse anti-VP2, and rabbit anti-EaSAG1 were prepared in our lab. Horseradish peroxidase (HRP)-conjugated goat-anti-mouse antibody was purchased from M&C Gene Technology Ltd. (Beijing, China). A monoclonal antibody, mouse anti-glyceraldehyde-3-phosphate dehydrogenase (GAPDH) (60004-1-Ig), was purchased from Proteintech Group, Inc.

### Plasmids construction

The coding sequence of *vp2* gene (1,359 bp) is from IBDV European strain Faragher 52/70 (sequence ID: D00869.2), codon-optimized (http://genomes.urv.es/OPTIMIZER/) according to codon usage bias in *E. acervulina* (http://www.kazusa.or.jp/codon/) and synthesized by Beijing Ruibiotech Co., Ltd (Beijing, China). Plasmids pEaMIC-VP2 and pEaSAG-VP2 were constructed as follows: (i) for pEaMIC-VP2 plasmid, full-length *vp2* gene was fused to 3´ end of EaMIC2 (gene ID: EAH_00000090) (34) by a flexible linker peptide (L, 42 bp), and ligated to the 5´ end of *eyfp* gene with a porcine teschovirus-1 2A peptide (P2A, 66 bp) (35), which were under the regulation of a MIC2 promoter and a 3′ regulatory sequence from *E. tenella* in the expression cassette; (ii) for pEaSAG-VP2 plasmid, *vp2* gene was fused between sequences encoding the signal and mature peptides of EaSAG1 (gene ID: EAH_00003690) with the flexible linker peptide at its 3´ end, under the regulation of a SAG1 promoter and a 3´ regulatory sequence of actin protein from *E. acervulina* in one expression cassette, which was ligated with another expression cassette containing the EYFP fused with a signal sequence of *E. acervulina* microneme adhesive repeat domain containing protein 1 (EaMCP1) (gene ID: EAH_00017570) at 5´ end. Meanwhile, backbone was acquired by linearizing the plasmid pSDEP2AHA1A (13) with restriction enzyme SnaB I (NEW ENGLAND BioLabs Inc.). Fragments were amplified using primers (Table S1) by Q5 High-Fidelity DNA Polymerase (New England BioLabs, Inc.) and ligated by Seamless Cloning and Assembly Kit (TransGen Biotech, Beijing, China). Plasmid DNA was purified using the PhasePrep EndoFree Maxi Kit (Aidlab Biotech, Beijing, China) as described by the manufacturer.

### Transfection and selection of transgenic parasites

Sporozoites were purified through a diethylaminoethyl-52 cellulose column after releasing from sporulated oocysts (36). The two new plasmids, linearized with restriction enzyme SnaB I (New England BioLabs, Inc.), were transfected into 1 × 10^7^ EacWT sporozoites by REMI as previously described (37). The transfected *E. acervulina* sporozoites (total of 1 × 10^7^) were inoculated into the wing vein (14) of three 1-week-old chicks. And the oocysts were collected from feces between day 5 and day 8 after infection. Then, the transgenic oocysts were selected by FACS, using the MoFlo Cell Sorter (Dako-Cytomation, Fort Collins, CO), then propagation of next generation. Following single-sporocyst isolation (38), 1 of 11 and 1 of 13 birds infected with single sporocyst were collected successfully for EaMIC-VP2 and EaSAG-VP2 clonal expansion, respectively, which were used for identification and animal experiments.

### DNA extraction and PCR amplification, RNA extraction, and RT-qPCR

gDNA was extracted from sporulated oocysts of EacWT, EaMIC-VP2, and EaSAG-VP2 as previously described (12). Then the gDNA was used as templates to amplify the fragment of gene *vp2*, with the primer pairs P1 (5′-ATGACAAACCTGCAGGATC-3′)/P2 (5′-TCTTCTAATAGCTCTAATA-3′), P3 (5′- ATGGCTCGCGCTTTTTCG-3′)/P2 (5′-TCTTCTAATAGCTCTAATA-3′), and P1 (5′-ATGACAAACCTGCAGGATC-3′)/P4 (5′-TCAAAGAAGAACAGCAGAGG-3′) for identification of transgenic parasites at DNA level. The gDNA of EacWT was used as negative control, and PCR was conducted with 2 × Taq PCR MasterMix (Aidlab Biotech, Beijing, China) as described by the manufacturer.

For RNA extraction, 1 × 10^7^ sporulated oocysts were suspended with 200 µL Trizol (Invitrogen, USA), and 100 µL 1 mm diameter glass beads were added in 1.5 mL centrifuge tubes. The tubes were put in tissue grinder (Hoder, China), and the solution was sheared repeatedly (run time: 180 s, interruption interval: 10 s, run times: 3, frequency: 65 Hz). Then, the samples were centrifuged at 10,000 rpm for 5 min. The supernatant was transferred to new 1.5 mL tubes and mixed with Trizol (for a total volume to 1 mL). Total RNA was extracted by the Trizol (Invitrogen, USA) method and converted to cDNA using HiScript III 1st Strand cDNA synthesis kit (1gDNA wiper) (Vazyme, China), as previously described (39). For qPCR (Applied Biosystems, Thermo Fisher, USA), Taq Pro Universal SYBR qPCR master mix (Vazyme) was used to quantify the transcriptional level of interest gene *vp2* with the primer pair VP2-F (5′-TGCGATAGCAGCGATAGACC-3′)/VP2-R (5′-TGGTACTGGCTGCTAAACTGG-3′). The *gapdh* was used as a loading control with the primer pair GAPDH-F (5′-TCTGCACATATCTGGCGGTG-3′)/GAPDH-R (5′-GGCTGGTATTCCTCGTGGTT-3′). Comparative CT (2^−ΔΔCT^) (39) was used to compare the transcriptional level of *vp2* between the transgenic strains and EacWT.

### Western blot

The extraction of soluble proteins from the oocyst with liquid nitrogen grinding method and Western blot followed protocols established previously (16). Briefly, proteins were separated on SDS-polyacrylamide gels and transferred onto polyvinylidene difluoride membranes, then probed with antibodies. The EacWT soluble protein and rVP2 protein expressed by the pET32a (0.5 mg/mL, 1: 10,000 dilution) were used as negative and positive control, respectively. Polyclonal mouse anti-VP2 (1:500 dilution) was the primary antibody prepared for testing. HRP-conjugated goat-anti-rabbit or goat-anti-mouse antibody (Macgene, 1:5,000 dilution) served as the secondary antibody. A monoclonal antibody, mouse anti-GAPDH (Proteintech, 1:5,000 dilution), was used to detect the GAPDH house-keeping protein (for gauging protein loading), respectively. The theoretical molecular weight of target proteins is VP2—49.83 kDa, EaSAG1—26.18 kDa, and EaMIC2—32.23 kDa, respectively.

### LC-MS/MS analysis

The extraction of soluble proteins of EacWT and the two transgenic *E. acervulina* parasites was done with liquid nitrogen grinding method as reported previously (13), then the samples were sent to Beijing Qinglian Biao Biotechnology Co., Ltd. (China) for LC-MS/MS identification.

### IFA

When detecting extracellular sporozoites, IFA was performed as previously (24). Briefly, the sporozoite suspension was applied onto a poly-l-lysine-coated slide, fixed in precooled carbinol, and then washed with PBS three times. Subsequently, treatment of the sporozoites with 0.2% Triton X-100 (Sigma-Aldrich, America) was done. Then, sporozoites were blocked with 1% bovine serum albumin before incubation with primary antibodies in a humidified chamber. After three washes with PBS, the slide was further incubated with secondary antibodies. After another three washes, 4’,6-diamidino-2’-phenylindole (DAPI) was used to label the nuclei of parasites for 5 min. When detecting intracellular sporozoites, freshly purified sporozoites were inoculated on HFF monolayers grown on glass coverslips in 12-well plates, and IFA was performed 24 hours post invasion as that done on tachyzoites of *Toxoplasma gondii* (40). Polyclonal antibodies (1:200 dilution) mouse anti-VP2 and rabbit anti-EaMIC2 or rabbit anti-EaSAG1 were used to identify the co-location of VP2 with EaMIC2 or EaSAG1 in transgenic parasites. The Cy3-conjugated goat anti-mouse IgG, Cy3-conjugated goat anti-rabbit IgG, and/or fluorescein isothiocyanate (FITC)-conjugated goat anti-rabbit IgG (Proteintech, USA, 1:200 dilution) was used as a secondary antibody. High-content imaging was performed with the confocal microscope (SP5, Leica, Germany).

### Reproductive capacity of transgenic *E. acervulina*

Nine 3-day-old AA broilers were divided randomly into three groups, i.e., EacWT, EaMIC-VP2, and EaSAG-VP2, in which each bird was inoculated orally with 200 sporulated oocysts, respectively. Fecal samples were collected every 24 h from 96 to 312 h post infection. The total oocysts output of each bird (N) was counted by McMaster chamber and calculated using the following formula: *N* = (*n* × 100 / 15 × w × a) ÷ 3 (*n*, average number of oocysts in the two compartments; w, weight of fecal samples; a, dilution ratio) (41).

### Immunological experiment

A universal immunization program was designed wherein 7-day-old (7d) SPF chickens were divided into six groups (Table 1). They received the first immunization at 7 days, the second one after a 14-day interval, and were then challenged with a 100-fold dose of live IBDV vaccine (B87 strain) following another 14-day interval. Serum samples were collected before every immunization and challenge. Bursal samples were collected on the 3 days, 7 days, and/or 14 days after challenge. The groups, immunization doses, and IBDV challenge are as follows (Table 1).

### Enzyme-linked immunosorbent assay (ELISA)

The ELISA was used to detect the specific antibody response to *E. acervulina* and VP2 after the initial and subsequent immunizations. The former ELISA was conducted as previously (24), which sAg of EacWT was used as the coating Ag to coat the 96-well microtiter plates (2 µg/mL). The latter was tested by two commercial ELISA kits: ProFLOK IBD PLUS Ab Test kit (Zoetis, France) and IDEXX IBD Ab Test kit (America) as described by manufacturer, respectively.

### H&E and IHC

Bursal samples collected 7 days and 14 days after challenge were fixed in 4% formaldehyde for paraffin sections (39). Bursal tissues were cut transversely for three successive sections: one was stained with H&E (39) for histopathological examination, the other two were detected by IHC staining (14) using Mouse two-step test Kit (Mouse Enhanced Polymer test system) (ZSGB-BIO, Beijing, China) as described by the manufacturer with the antibody anti-VP2 or PBS (negative control) for confirming whether the tissue damage was caused by IBDV challenge. Histopathological examination was scanned by digital pathology slide scanner (Rocha, Switzerland) and scored bursal lesion (42).

### Statistical analysis

All statistical analyses were performed in GraphPad Prism 8.0.1 software: two-way analysis of variance (ANOVA) was used to analyze the dynamic change of oocyst output in the curve and antibody titer; one-way ANOVA was used to analyze the total oocyst output in 5–8 days after inoculation; multiple *t*-test was used to analyze the BBW.

## Data Availability

The mass spectrometry proteomics data have been deposited to the ProteomeXchange Consortium (http://proteomecentral.proteomexchange.org) via the iProX partner repository (43, 44) with the data set identifier PXD046252. Other data and materials are all present in the contents of the article.

## SUPPLEMENTAL MATERIAL

Supplemental figures iai.00456-23-s0001.pdf Fig. S1 to Fig. S4.

Table S1 iai.00456-23-s0002.xlsx Primers used for plasmid construction.

ASM does not own the copyrights to Supplemental Material that may be linked to, or accessed through, an article. The authors have granted ASM a non-exclusive, world-wide license to publish the Supplemental Material files. Please contact the corresponding author directly for reuse.
