# Supplementary material for: Microneme-located VP2 in Eimeria acervulina elicits effective protective immunity against infectious bursal disease virus
Source: Infect Immun. 2024 Jan 5;92(2):e00456-23. doi: 10.1128/iai.00456-23 (PMC10863409; doi:10.1128/iai.00456-23)
Supplement: Supplemental figures — Fig. S1 to Fig. S4. [file iai.00456-23-s0001.pdf]

SUPPLEMENTARY FIGURES

Additional file 1: Figure S1. Antibody titers against IBDV in serum after immunization and bursal index after challenge.

Additional file 2: Figure S2. H&E staining of bursal sections after IBDV challenge.

Additional file 3: Figure S3. VP2 peptides detected by liquid chromatogram-mass spectrometry (LC-MS) in transgenic *Eimeria*.

Additional file 4: Figure S4. Antibody titers against IBDV in SPF chicken serum using the ProFLOK^{TM} IBD PLUS Ab Test kit.

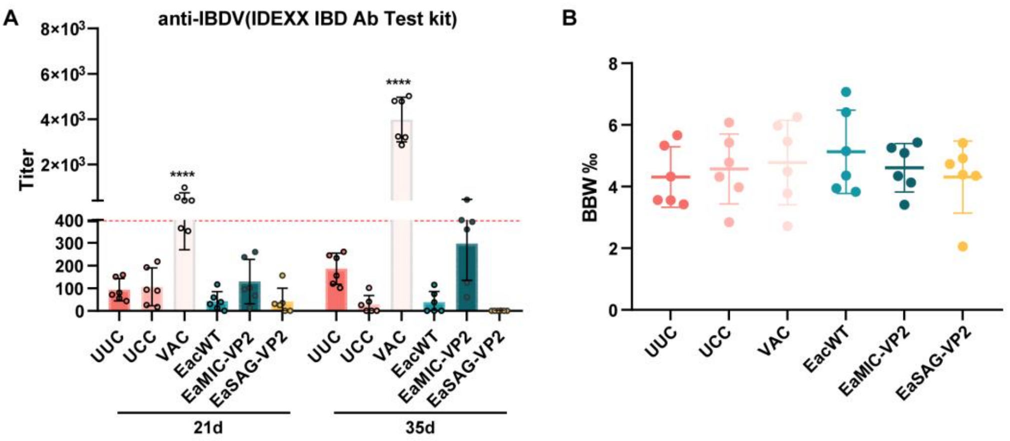

Figure S1. Antibody titers against IBDV in serum after immunization and bursal index after challenge. **(A)** Antibody titers against IBDV detected by IDEXX IBD Ab Test kit. The red dashed line represents a titer equal to 396 (E), values greater than which are considered positive. **(B)** BBW at 3 days after challenge. BBW = bursal weight (in grams) / body weight (in grams) *1000. Asterisks represent for statistic difference: * stands for p<0.05, ** stands for p<0.01, *** stands for p<0.001, **** stands for p<0.0001.

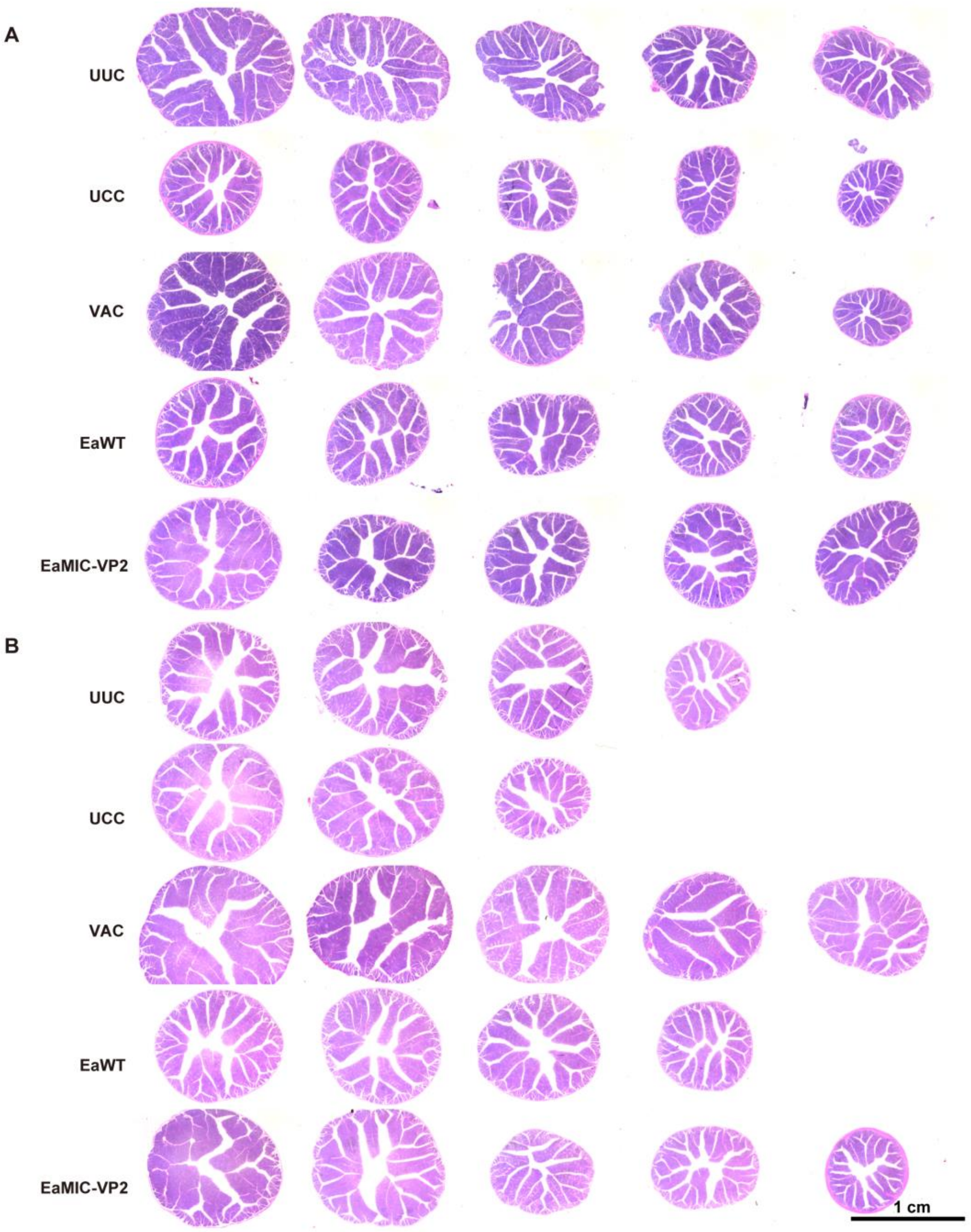

Figure S2. H&E staining of bursal sections after IBDV challenge. H&E staining of bursal sections at 7 days **(A)** and 14 days **(B)** after IBDV challenge. 0.63 × 9.4

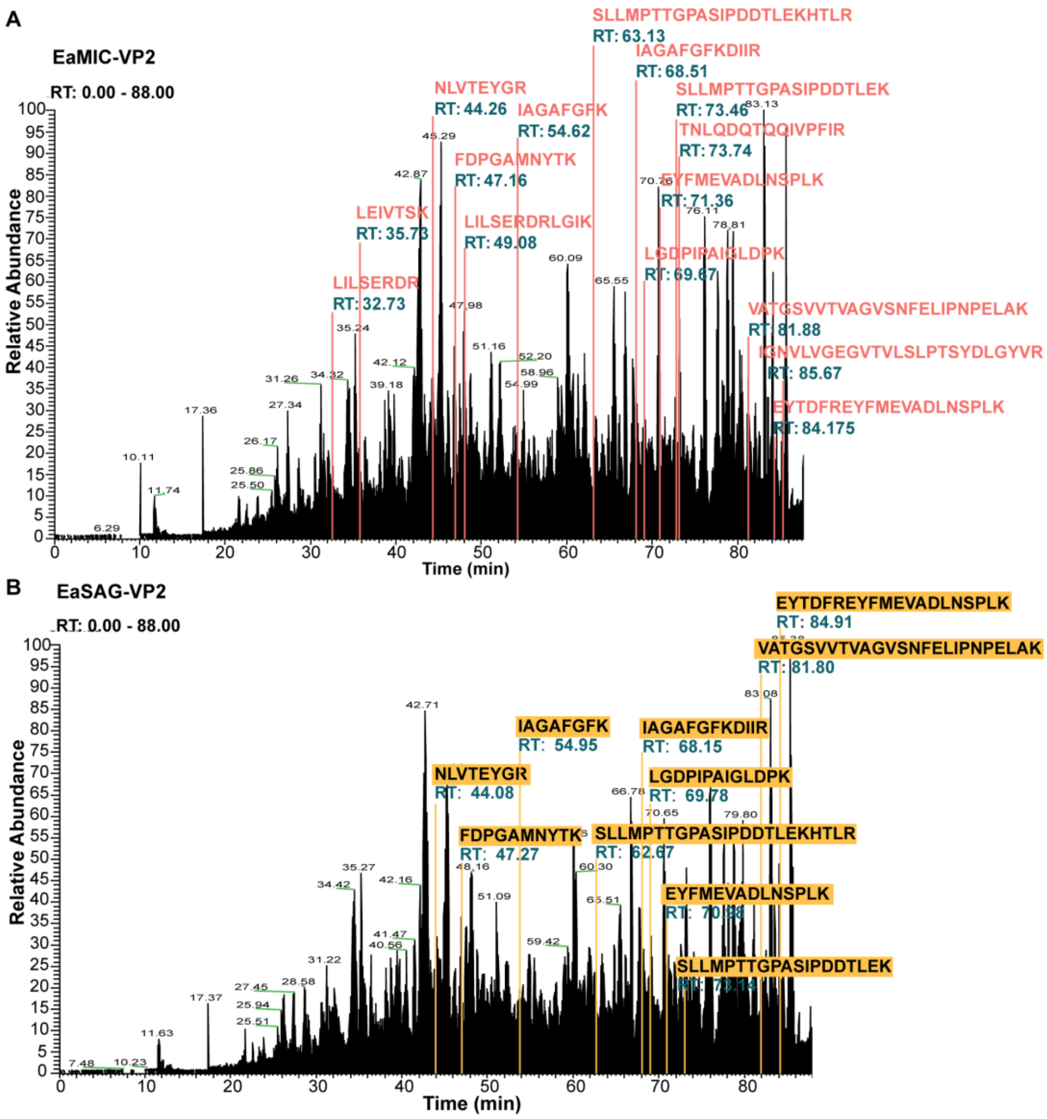

C

MTNLQDQTQQIVPFIRSLLMPTTGPASIPDDTLEKHTLRSETSTYNLTVGDTGSGLIVFFPGFPGSIVGAHYTLQSNGNY
KFDQMLLTAQNLPASYNYCRLVSRSLTVRSSTLPGGVYALNGTINAVTFQGSLSELTDVSYNGLMSATANINDKIGNVLV
GEGVTVLSLPTSYDLGYVRLGDPIPAIGLDPKMVATCDSSDRPRVYTITAADDYQFSSQYQPGGVTITLFSANIDAITSL
SIGGELVFQTSVQGLVLGATIYLIGFDGTAVITRAVAADNGLTAGTDNLMPFNLVIPTNEITQPITSIKLEIVTSKSGGQ
AGDQMSWSASGSLAVTIHGGNYPGALRPVTLVAYERVATGSVVTVAGVSNFELIPNPELAKNLVTEYGRFDPGAMNYTKL
ILSERDRLGIKTVWPTREYTDFREYFMEVADLNSPLKIAGAFGFKDIIRAIRR

Figure S3. VP2 peptides detected by liquid chromatogram-mass spectrometry (LC-MS) in transgenic *Eimeria*. (**A**) Secondary mass spectrum of VP2 peptides detected in soluble antigen of EaMIC-VP2 parasites. (**B**) Secondary mass spectrum of VP2 peptides detected in soluble antigen of EaSAG-VP2 parasites. (**C**) VP2 amino acid sequence. Pink letters were VP2 peptide sequences identified from EaMIC-VP2, and yellow highlighted parts were from EaSAG-VP2. RT: retention time.

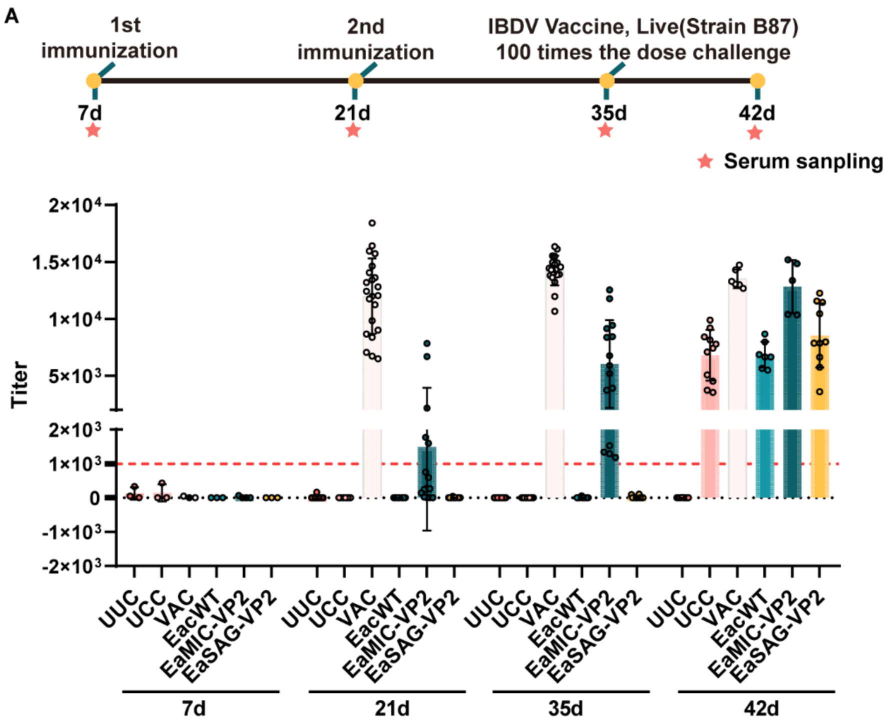

Figure S4. Antibody titers against IBDV in SPF chicken serum using the ProFLOK^{TM} IBD PLUS Ab Test kit. The immunological experiment was performed on SPF chickens according to Table 1 but the dose of oocysts in 1st immunization increased to 2×10^4.
